# Adverse pregnancy outcomes and complications of tuberculosis in pregnant women

**DOI:** 10.3389/fcimb.2025.1550430

**Published:** 2025-10-02

**Authors:** Yinfeng Su, Xingxing Kong, Zhanpeng Chen, Xiaomin Wang, Shuihua Lu

**Affiliations:** ^1^ Department of Infectious Diseases, Affiliated Hospital of Southwest Medical University, Luzhou, Sichuan, China; ^2^ National Clinical Research Center for Infectious Diseases, Shenzhen Clinical Research Center for Tuberculosis, Shenzhen Third People’s Hospital, Shenzhen, Guangdong, China

**Keywords:** *Mycobacterium tuberculosis*, tuberculosis, pulmonary TB, pregnancy outcome, pregnancy complication

## Abstract

Tuberculosis in pregnancy is a series of diseases caused by *Mycobacterium tuberculosis* (*Mtb*). Exact global incidence rates are difficult to obtain and are expected to be similar to those in the general population, with potentially higher rates in developing countries. Tuberculosis in pregnancy is associated with adverse reactions in both mother and fetus, but the mechanism is not fully understood. Although many studies have explored the impact of tuberculosis on pregnancy outcomes and complications, there are still relatively few summary articles on it. This review aims to briefly summarize the adverse pregnancy outcomes and related complications of pregnant women with tuberculosis. Studies have shown that the risk of death for mothers with tuberculosis increases, and these mothers are more likely to have complications such as gestational hypertension, rupture of pulmonary bullae, postpartum hemorrhage, sepsis, anemia, placental chorioamnionitis, and gestational diabetes. In addition, tuberculosis infection during pregnancy also increases the risk of vertical transmission of Human Immunodeficiency Virus (HIV), leading to an increased chance of fetal infection with *Mtb* and an increased possibility of congenital tuberculosis in newborns. Besides, fetuses born to mothers with tuberculosis are at an increased risk of congenital malformations, intrauterine growth restriction, low birth weight, premature birth, miscarriage, and stillbirth.

## Graphical Abstract

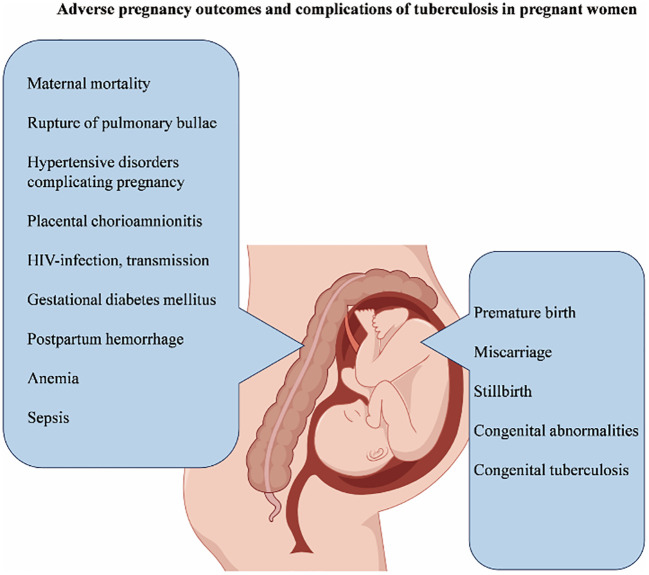

## 1 Introduction

Pregnancy with tuberculosis (PWT) is caused by an infection with *Mycobacterium tuberculosis* (*Mtb*), which can occur either during pregnancy or when Tuberculosis (TB) remains untreated during that time (Phoswa et al., 2020; Kgathi and Phoswa, 2021). TB is a key risk factor for maternal mortality, contributing to 6–15% of all maternal mortality (Tohme et al., 2024; WHO, 2024), with the highest mortality rate among pregnant women co-infected with Human Immunodeficiency Virus (HIV) (Bekker et al., 2016). Accurate data on the incidence of TB during pregnancy are difficult to obtain in many countries and regions due to a variety of factors. Loto OM (Loto and Awowole, 2012) claimed that the incidence of TB among pregnant women is similar to that in the general population and could be higher in developing countries. Sugarman et al. (2014) estimated that 216500 (95% uncertainty range 192 100-247 000) active tuberculosis cases existed in pregnant women globally in 2011. The greatest burdens were in the WHO African region and South East Asian region. According to the 2019 Global Conference on Tuberculosis and Lung Disease reported, approximately 200,000 pregnant or postpartum women develop tuberculosis annually (comprising 151,000 pregnant and 49,000 postpartum cases) (Mafirakureva, 2019).

**Figure 1:**
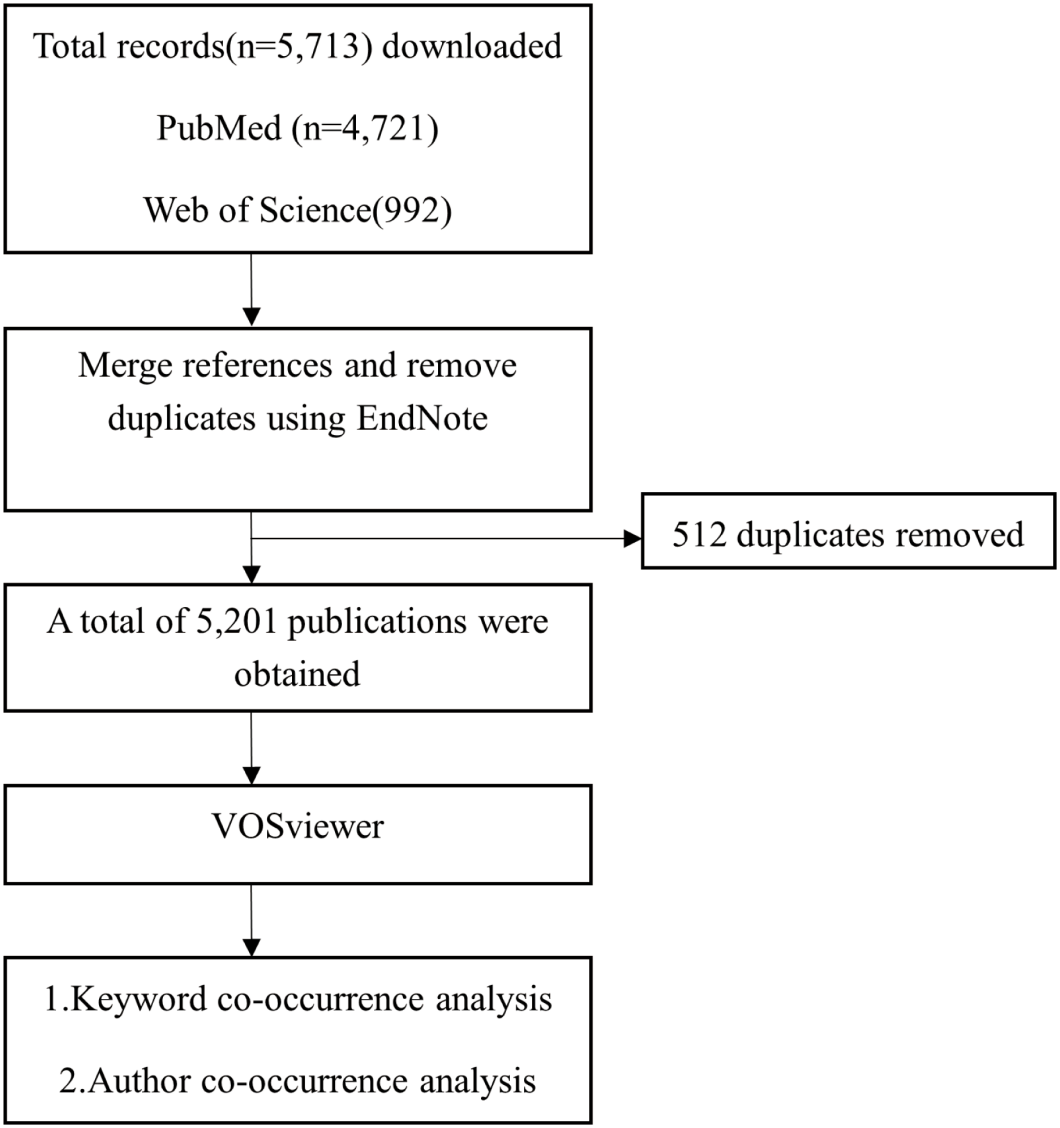
Flowchart of the VOSviewer analysis process. Records on pregnancy and tuberculosis were retrieved from PubMed (n = 4,721) and Web of Science (n = 992). These were then merged in EndNote X8 and de-duplicated (512 duplicates removed), yielding 5,201 unique publications. These records were exported in RIS format and analyzed in VOSviewer using defined threshold settings to perform keyword and author co-occurrence analyses.

**Figure 2:**
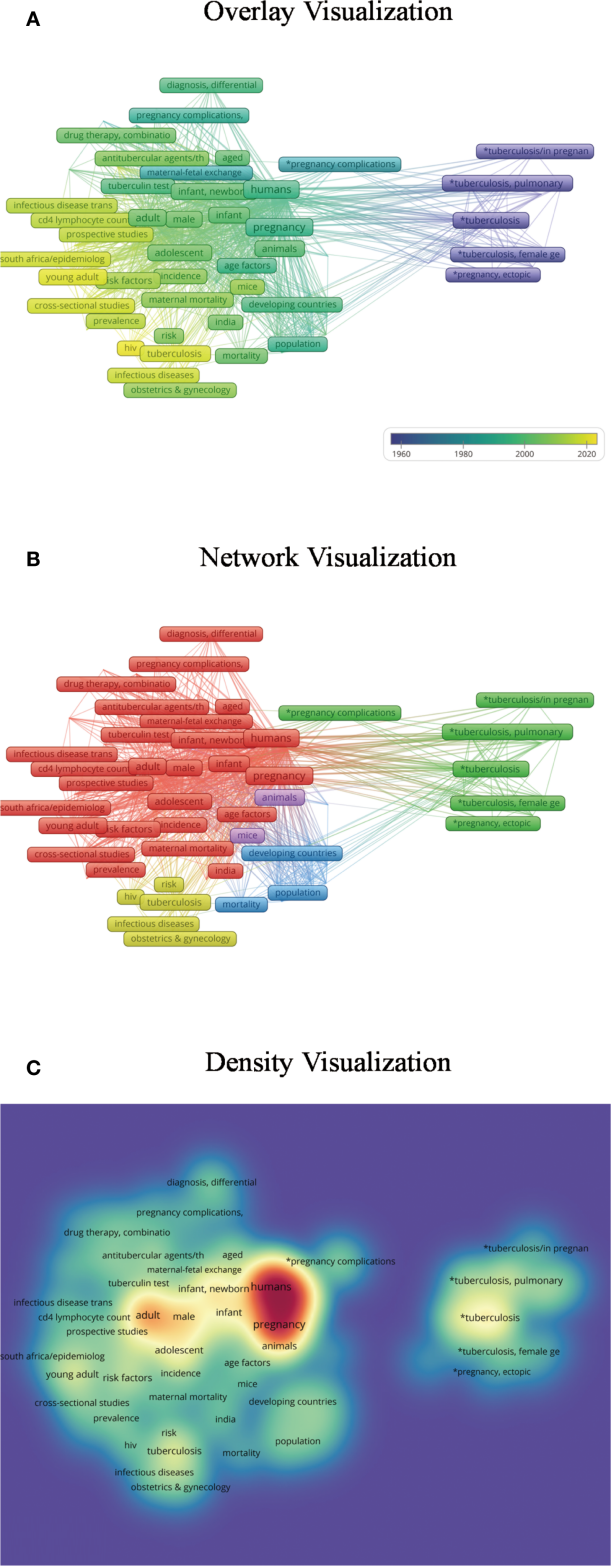
Keyword co-occurrence analysis. **(A)** The overlay visualization reveals that research on tuberculosis in pregnancy initially focused on genital tuberculosis, later expanding to anti-tuberculosis treatment and diverse social, demographic, and economic factors. Complications and adverse pregnancy outcomes have remained a consistent focus, while co-infection with HIV during pregnancy has emerged as a recent research hotspot. In this visualization, darker colors represent keywords with earlier appearance, whereas lighter colors denote more recent topics. **(B)** The network visualization categorizes publications into five clusters: (1) genital tuberculosis and pregnancy complications; (2) diagnosis, treatment, prevention, and risk factors; (3) HIV co-infection; (4) socioeconomic and demographic influences across various countries; and (5) relevant animal studies. Nodes of the same color belong to related thematic categories. **(C)** In the density visualization, keyword frequency is reflected by color intensity, with darker areas indicating higher occurrence rates.

**Figure 3:**
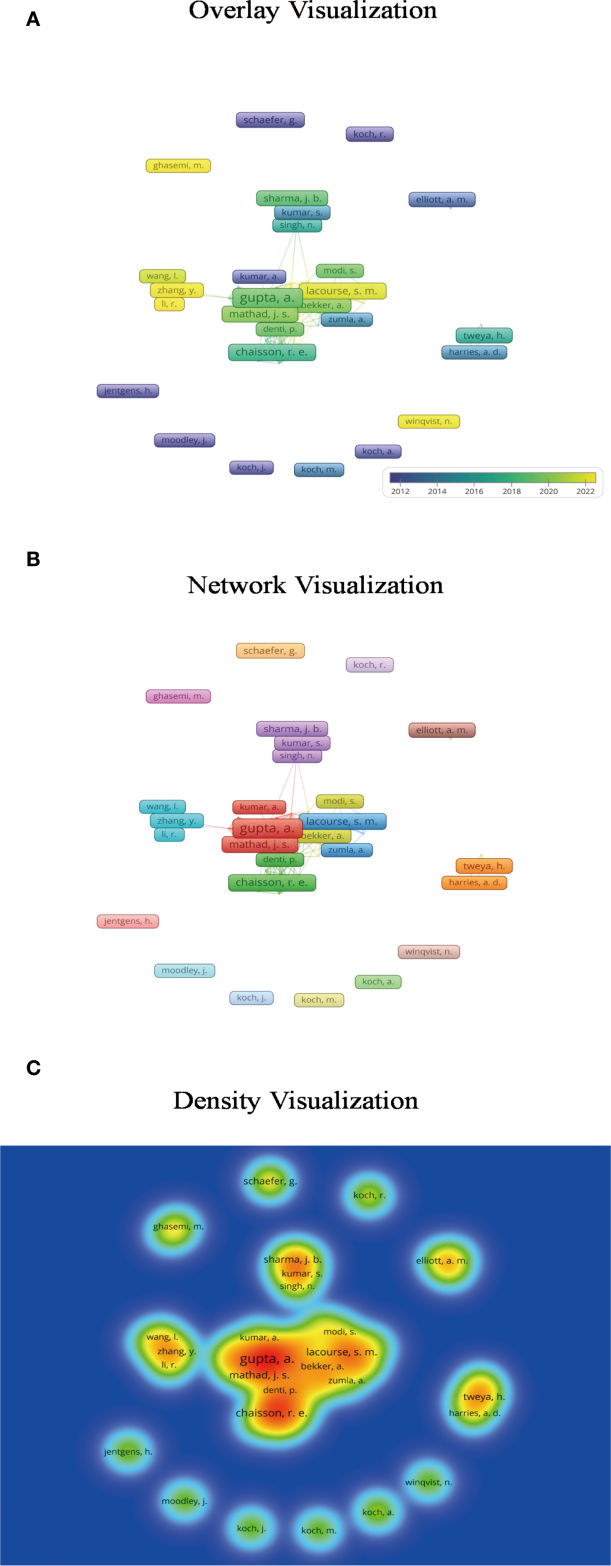
Author co-occurrence analysis. **(A)** The overlay visualization indicates that early investigations in this field were initiated by researchers such as Koch, whereas significant contributions from Chinese scholars have emerged only in recent years. Darker colors represent earlier publication years, while lighter shades indicate more recent years of publication. **(B)** Based on an appropriate threshold, the network visualization identifies 17 distinct author clusters, each represented by a unique color. Authors within the same cluster belong to the same research community and exhibit collaborative relationships. **(C)** The density visualization highlights that authors such as Gupta, Chaisson, and Mathad serve as central contributors in the field of tuberculosis in pregnancy research. In this visualization, brighter colors indicate a higher publication output and greater influence of an author.

The author collected literature from PubMed and Web of Science and conducted a keyword clustering analysis and co-authorship analysis with VOSviewer (**Figures 1**–**3**) (van Eck and Waltman, 2010). Pregnancy complications emerged as a prominent theme in the analysis and remain a consistent research focus. This review synthesizes evidence on adverse pregnancy outcomes and complications in pregnant women with active drug-sensitive or drug-resistant tuberculosis (TB). It summarizes outcome disparities between TB-affected and non-affected pregnancies and characterizes complications attributable to anti-TB therapy. Given the inconsistent definitions of pregnancy outcomes and complications across studies, we classified maternal death and infant outcomes as adverse pregnancy outcomes, while concurrent maternal diseases are defined as complications.

## 2 The impact of pregnancy on tuberculosis susceptibility

There is a view that pregnancy itself does not increase susceptibility to tuberculosis or affect its course, and the factors influencing tuberculosis include physiological changes during pregnancy (neuroendocrine regulation, weaker immune system), as well as atypical symptoms or masking by pregnancy, which lead to delayed diagnosis of tuberculosis during pregnancy and thus increase the risk of progression to active tuberculosis (Pazos et al., 2012; Gould and Aronoff, 2016; Phoswa et al., 2020; Hui and Lao, 2022). Others believe that pregnancy increases susceptibility to tuberculosis, doubling or tripling the risk of tuberculosis (Snow et al., 2020). Multiple gestations further increase the risk of reactivation of latent tuberculosis (Phoswa et al., 2020). Pregnant women may be like the general population, in whom progression to active tuberculosis is influenced by smoking, alcohol consumption, malnutrition, close contact with untreated sputum smear-positive individuals, diabetes mellitus, and HIV infection (Phoswa et al., 2020).

## 3 Tuberculosis and adverse pregnancy outcomes

### 3.1 Tuberculosis and maternal mortality, premature birth, miscarriage, stillbirth

A retrospective study in the United States found that pregnant women with tuberculosis (TB) primarily belong to the 25–34 age group, with a significant prevalence among Hispanic women. Hospitalized pregnant women with tuberculosis had a markedly higher maternal mortality rate and an increased rate of premature births compared to those without TB. Unfortunately, the study did not specify whether the rates of miscarriage and stillbirth were also elevated. Additionally, the study indicated women with PWT had a higher prevalence of co-infection with HIV, and those hospitalized for delivery were more likely to experience complications such as chorioamnionitis, preterm birth, postpartum anemia, blood transfusions, pneumonia, acute respiratory distress syndrome, mechanical ventilation, and congenital abnormalities in infants (El-Messidi et al., 2016). Sobhy et al. (2017) published a systematic review on the outcomes of tuberculosis during pregnancy and postpartum, which included 13 studies involving 3,384 women with active tuberculosis and 119,448 non-tuberculous pregnant women. The study found that pregnant women with active tuberculosis had poorer perinatal outcomes, with a nearly threefold rise in mortality risk (OR=2.8, 95% CI 1.7-4.6), a fourfold increase in neonatal perinatal mortality risk (OR=4.2, 95% CI 1.5-11.8), a 1.7-fold higher rate of preterm births (OR=1.7, 95% CI 1.2–2.4) and a 9-fold increased likelihood of miscarriage (OR=9.0, 95% CI 4.93-16.67). Furthermore, the study highlighted that pregnant women with active TB face increased risks of anemia, cesarean delivery, fetal respiratory distress, low birth weight, and congenital malformations in infants.

Snow et al. (2020) conducted a meta-analysis of review articles published after 2010 regarding tuberculosis in pregnant women and newborns, and reaffirmed that tuberculosis during pregnancy elevates the risks of maternal miscarriage, maternal mortality, and neonatal death. Nguyen et al. (2022) noted that pregnant women with tuberculosis are at an increased risk for adverse fetal outcomes (such as low birth weight, preterm birth, and miscarriage) as well as maternal complications (including anemia, preeclampsia, and death). And the study have recommended implementing comprehensive family planning for both drug-sensitive and drug-resistant tuberculosis patients to reduce the risk of adverse pregnancy outcomes. This comprehensive family planning should include providing counseling on pregnancy-related risks and safe contraceptive methods, as well as appropriate care if women wish to conceive during treatment. Notably, Women with multidrug-resistant tuberculosis (MDR-TB) during pregnancy demonstrated a substantially elevated risk of adverse pregnancy outcomes. Furthermore, increased incidences of anti-TB treatment-related adverse events including hepatic impairment, renal dysfunction, hearing loss, and hypokalemia are observed. However, following effective anti-TB therapy, both treatment success rates and pregnancy outcomes significantly improved (Alene et al., 2021; Mokhele et al., 2021; Alene et al., 2022; Crocker-Buque et al., 2024; Lotia Farrukh et al., 2024). Moreover, Meehan et al. (2024) identified advanced maternal age (35–44 vs. 25–34 years), HIV co-infection, unsuccessful TB treatment outcomes, and late antenatal care initiation (≤1 month pre-delivery) as significant predictors of adverse pregnancy outcomes (Maternal Mortality, Premature Birth, Miscarriage, Stillbirth).

These findings suggested that women with tuberculosis during pregnancy faced higher risks of maternal mortality and adverse infant outcomes, such as premature birth, miscarriage, and stillbirth, compared to those without tuberculosis. It is recommended that family planning services for these women be strengthened.

### 3.2 Tuberculosis and congenital abnormalities in infants

Numerous studies have explored the relationship between tuberculosis (TB) and pregnancy outcomes, with a specific focus on congenital abnormalities. A large retrospective cohort study in the United States indicated that infants born to mothers with TB have a higher incidence of congenital defects (El-Messidi et al., 2016). Sobhy et al. (2017) found that pregnant women with active TB are at an increased risk of giving birth to infants with congenital malformations, but this association did not reach statistical significance(OR 3.4, 95% CI 0.71–16.7; I2 = 73%) Similarly, Pranevicius et al. (2003) observed a rise in neonatal developmental abnormalities among pregnant women with TB, including conditions such as spina bifida and congenital hip dislocation. Methodologically regrettable is that a considerable number of studies merely assessed the risk of congenital malformations among offspring of tuberculosis (TB)-affected pregnancies compared to controls, yet failed to specify the types of anomalies.

Besides, the use of various anti-TB drugs during pregnancy may increase the risk of congenital anomalies. For example, streptomycin has been confirmed to have teratogenic effects, with approximately 1 in 6 infants experiencing hearing or balance issues, indicating that its use during pregnancy should be avoided (Bothamley, 2001). A 2015 case report documented a pregnant woman with pulmonary tuberculosis who received rifampicin during the first trimester, subsequently delivering a male neonate with a hypoplastic right forearm (Kalayci et al., 2015). And One study reported that among 196 HIV-positive pregnant women exposed to isoniazid preventive therapy(IPT) around the time of conception, one delivered an infant with talipes equinovarus (Taylor et al., 2013). Although a small-scale case-control study did not observe teratogenic risks associated with oral anti-tuberculous drugs (Czeizel et al., 2001), its limited sample size raises concerns about its reliability. A recent meta-analysis showed that the prevalence of congenital anomalies in anti-TB drugs used during pregnancy was 1.9% (95% CI: 0.07–5.1%, I^2^=0.0%, P=0.404). According to the drug category, the prevalence of congenital anomalies was 1.6% for isoniazid and 3.2% for isoniazid plus rifapentine (Wu et al., 2023). Additionally, both fluoroquinolones and ethionamide belong to Category C medications: the former may damage the fetal articular cartilage, increasing the risk of juvenile arthritis and arthropathies; the latter, as a second-line drug for treating tuberculosis, has been proven to cause teratogenicity when used at high doses in rodents. Therefore, neither of these two drugs is advisable for use during pregnancy (Arbex et al., 2010; Gee et al., 2014).

In summary, pregnant women with tuberculosis may experience fetal developmental abnormalities, increasing the proportion of infants with congenital defects. Therefore, clinicians must exercise caution in selecting anti-TB drugs avoiding the use of known teratogenic drugs. For drug-sensitive pregnancy tuberculosis, the standard 6-month 2HRZE/4HR regimen remains the first choice due to its low teratogenicity, with therapeutic benefits far outweighing the risks of untreated tuberculosis (WHO, 2022).

### 3.3 Tuberculosis and congenital tuberculosis in infants

It is possible for an infant be infected with tuberculosis (TB) through vertical transmission from a mother with TB or from a person in the household with active pulmonary TB. when the infection is acquired through vertical transmission from the mother, this is referred to as congenital tuberculosis. Congenital tuberculosis occurs through three primary mechanisms: (1) transplacental hematogenous spread via the umbilical vein, (2) aspiration of infected amniotic fluid or vaginal secretions, (3) direct inoculation during vaginal delivery (Hu et al., 2023; Lee et al., 2024). It is worth noting that although congenital tuberculosis is extremely rare (Shao et al., 2021), it has drawn increasing attention in recent years due to its potential to cause preterm birth in infants (70/170 cases), early onset of disease (within 20 days), high mortality rate (68/169 cases), a high proportion of miliary tuberculosis (83/170 cases), and nonspecific symptoms and signs (all 170 cases) (Peng et al., 2011). congenital tuberculosis can lead to primary foci in the liver, lungs, and gastrointestinal tract, which is notably different from adult primary TB, where approximately 80% of infectious foci are localized in the lungs (Adhikari, 2009; Li et al., 2019; Hirsch-Moverman et al., 2024).

According to the diagnostic criteria established by Cantwell in 1994, the diagnosis of congenital TB requires the following: 1) evidence of tuberculous lesions; and 2) at least one of the following: lesions within the first week of life, a primary hepatic complex or caseating hepatic granulomas, infection of the placenta or maternal reproductive tract, or thorough investigation to exclude the possibility of postpartum transmission (Cantwell et al., 1994). It is notable that women diagnosed with placental tuberculosis following *in vitro* fertilization have a higher risk of congenital tuberculosis in their infants (3 in 10) (Hu et al., 2023).

For infants with drug-sensitive congenital tuberculosis, standardized treatment includes isoniazid (10–15 mg/kg/day), rifampicin (10–20 mg/kg/day), pyrazinamide (15–30 mg/kg/day), and ethambutol (15–25 mg/kg/day), with isoniazid and rifampicin continued for 4–10 months based on severity (Li et al., 2019; Lee et al., 2024). Even if congenital tuberculosis is ruled out, fetuses born to mothers with drug-sensitive tuberculosis require isoniazid (10 mg/kg) prophylaxis for 6 months regardless of tuberculin test results, with regular symptom monitoring (Shao et al., 2021; Lee et al., 2024). Additionally, rifampicin or levofloxacin may be considered for window prophylaxis in other newborns exposed to isoniazid-resistant congenital tuberculosis infants, including preterm infants.

In conclusion, Pregnant women with concurrent TB increase the risk of vertical transmission of *Mtb*. Additionally, breastfeeding by mothers who have not received prenatal care and who have pulmonary TB or smear-positive tuberculosis, also elevates the risk of mother-to-child transmission (Adhikari, 2009; Aziz and Suryawan, 2023). Although congenital tuberculosis is rare, it imposes substantial hazards on infants.

## 4 Complications in pregnant women with tuberculosis and its management

### 4.1 Tuberculosis and rupture of pulmonary bullae during pregnancy

Chronic pulmonary TB can lead to lung scarring and permanent damage, resulting in the formation of pulmonary bullae. Pulmonary bullae are defined as air-filled spaces with a diameter greater than 1cm and wall thickness less than or equal to 1mm, potentially occupying more than 30% of the lung. Large pulmonary bullae may present clinically with symptoms such as cough, chest pain, and dyspnea, but may also be asymptomatic in some cases. Bullous lung disease due to tuberculosis is more commonly observed in pediatric patients and is rare in adults. While pulmonary bullae are generally benign, there is an increased the risk of rupture during pregnancy, posing a significant threat to the patient’s life. During labor, actions such as the Valsalva maneuver, increased alveolar ventilation, and positive pressure ventilation during general anesthesia can greatly elevate the risk of bullae rupture, leading to secondary pneumothorax. Severe cases may result in tension pneumothorax, bronchopleural fistula, and acute respiratory failure, thereby increasing the risk of the need for mechanical ventilation. To reduce the risk of bullae rupture during delivery in pregnant women with tuberculosis, vacuum extraction is recommended to shorten the second stage of labor, minimizing the need for Valsalva maneuvers and avoiding increases in intrapulmonary pressure (Aziz and Suryawan, 2023; Morán-Mariños et al., 2024).

In conclusion, pulmonary bullae may be benign under normal conditions. However, in pregnant patients, physiological changes during pregnancy (such as increased abdominal pressure and diaphragmatic elevation) may increase the risk of pneumothorax. When combined with underlying lung lesions caused by tuberculosis, the management of bullae rupture becomes more challenging, potentially threatening the safety of both the mother and the fetus.

### 4.2 Tuberculosis and hypertensive disorders complicating pregnancy

One out of every ten women develops hypertensive disorders before childbirth (Gynecologists, 2013). Hypertensive disorders of pregnancy, especially preeclampsia and eclampsia, are major causes of acute morbidity, long-term disability, and death in both mothers and infants. The aim of treatment is to prevent severe preeclampsia and eclampsia, reduce perinatal complications and maternal and infant mortality, and improve perinatal prognosis (Abalos et al., 2018). Timely termination of pregnancy is a crucial treatment for preeclampsia and eclampsia, and magnesium sulfate is the first-choice drug for treating eclampsia and preventing the recurrence of convulsions (Yu and Zhou, 2022).

A 2024 meta-analysis that included 16 surveys from Asia and Africa (with a total sample size of n=740,815) found significant differences in the prevalence of hypertension between non-tuberculosis family members living in households with at least one tuberculosis patient and those without (Hamada et al., 2024). Also, Phoswa et al. (2020) suggested that women with tuberculosis infection have a higher risk of developing hypertensive disorders in pregnancy compared with non-tuberculosis patients. The odds ratio (OR) for severe preeclampsia is 1.7, and that for eclampsia is 3.9. The risk is even higher in cases of tuberculosis-HIV co-infection. It is hypothesized that the reason for this may be related to the overactivation of angiogenic factors (such as vascular endothelial growth factor [VEGF], nitric oxide [NO], angiotensin 2 [Ang 2], and intracellular adhesion molecule [ICAM]) and inflammatory cytokines (such as interleukin-2 [IL-2], IL-17, and interferon-γ [INF-γ]) (Dennis et al., 2018; Phoswa et al., 2020).

A meta-analysis included 63 trials indicated that pharmacological antihypertensive treatment in pregnant women with mild to moderate hypertension can reduce the risk of developing severe hypertension, but its impact on other clinical outcomes (such as maternal death, severe preeclampsia, eclampsia, fetal miscarriage, neonatal stillbirth, and preterm birth) remains unclear (Abalos et al., 2018). Unfortunately, no research on gestational hypertension in pregnant individuals with tuberculosis has been identified. A multicenter retrospective study recommended postponing isoniazid treatment for latent tuberculosis until postpartum when feasible, and giving priority to the use of beta-blockers or calcium channel blockers rather than methyldopa when managing hypertension during pregnancy (Masood et al., 2024). Moreover, some studies have pointed out that beta-blockers and calcium channel blockers are highly effective in preventing severe hypertension (Abalos et al., 2018). Meanwhile, the combination of nifedipine tablets and magnesium sulfate in the treatment of pregnancy-induced hypertension syndrome (PIH) can improve coagulation function, weaken oxidative stress damage, and regulate the levels of serum endothelin-1 (ET-1) and nitric oxide (NO), thereby enhancing clinical efficacy (Yu and Zhou, 2022). However, rifampicin, as a first-line antituberculosis drug, can significantly reduce the efficacy of antihypertensive drugs such as calcium channel blockers, beta-blockers, and diuretics by inducing cytochrome P450 to accelerate the metabolism and excretion of antihypertensive drugs (Yogesh et al., 2023).

To summarize, women infected with tuberculosis have a higher risk of developing hypertensive disorders in pregnancy compared with those who are not infected. The treatment goal of hypertensive disorders in pregnancy is to decrease blood pressure in a timely manner, prevent the occurrence of severe preeclampsia and eclampsia, reduce perinatal complications and mortality rates of mothers and infants, and improve perinatal outcomes. Therefore, it is recommended to use beta-blockers or calcium channel blockers for hypertension management during pregnancy. In pregnant women with tuberculosis and gestational hypertension, concurrent rifampicin-based antitubercular therapy and beta-blockers/calcium channel blockers for antihypertension require close monitoring of antihypertensive plasma concentrations to enable timely dosage adjustments for optimal blood pressure control. If the patient’s condition progresses to preeclampsia or eclampsia, magnesium sulfate should be actively used for treatment, and pregnancy should be terminated in a timely manner according to the patient’s condition.

### 4.3 Tuberculosis and chorioamnionitis

Acute chorioamnionitis refers to acute inflammation of the chorion, which commonly occurs in ascending bacterial infections, while tuberculosis can also be a causative agent. A U.S survey indicated that the risk of chorioamnionitis in hospitalized patients with tuberculosis during childbirth is significantly higher than that in non-tuberculosis patients (El-Messidi et al., 2016). There has been a reported case of intrauterine tuberculosis presenting as acute chorioamnionitis in a 28-week-pregnant woman, and the delivered fetus was later confirmed to have congenital tuberculosis (Taweevisit et al., 2015). For women with tuberculosis and a history of cervical resection, the risk of placental chorioamnionitis during pregnancy is significantly increased (Romero et al., 2021; Kacerovsky et al., 2023). In addition, the risk of chorioamnionitis in HIV patients is also high (Mola, 1996; Maswime et al., 2021), predicting that the risk of acute chorioamnionitis in pregnant women with AIDS-tuberculosis coinfection is greatly increased. In Acute chorioamnionitis caused by tuberculosis infection, placental immunohistochemistry may show acute abscess-like inflammatory responses due to myeloperoxidase and CD68-positive neutrophils and histiocytes, accompanied by abundant acid-fast mycobacteria (Abramowsky et al., 2012; Bruce-Brand et al., 2021). Patients with clinical chorioamnionitis should receive intrapartum antibiotic and acetaminophen antipyretic treatments. The antibiotic treatment regimen should be formulated according to the specific bacterial infections to prevent adverse perinatal outcomes. The standard treatment for term clinical chorioamnionitis is the administration of antibiotics and induction of labor (Jung et al., 2024). For a pregnant woman with tuberculosis and confirmed chorioamnionitis, in addition to vigilance for fetal preterm birth, the possibility of congenital tuberculosis in the infant should also be monitored (Jain et al., 2022).

### 4.4 Tuberculosis, pregnancy and HIV

Tuberculosis is a major cause of morbidity and mortality among HIV-infected individuals living in high-burden, low-income, and middle-income countries (WHO, 2021a). Among tuberculosis patients, women are prone to concurrent HIV infection Moreover, the risk of tuberculosis infection for HIV-infected individuals during pregnancy also increases significantly. Studies have shown that the number of pregnant women with tuberculosis who are concurrently infected with HIV is significantly higher than that of pregnant women without tuberculosis (El-Messidi et al., 2016). Both HIV and pregnancy can lead to immunosuppression, further elevating the risk of progression to active tuberculosis. In addition, the risk of various extrapulmonary tuberculosis (such as tuberculous meningitis, tuberculous optic neuritis, etc.) among HIV-infected individuals also rises significantly (Garg and Sinha, 2011; Ismail et al., 2024).

Tuberculosis can not only increase the transmission of HIV but also utilize molecular mechanisms such as IL-2, IL-4, IFN-γ, TNF-α, and MCP-1 to increase HIV replication by activating viral replication in lymphocytes, monocytes, and macrophages at the site of *Mtb* infection (Collins et al., 2002; Vidya Vijayan et al., 2017). For pregnant women, concurrent infection with HIV and tuberculosis may exacerbate the vertical transmission of HIV from the mother to the fetus.

Patients with HIV-tuberculosis often face challenges in accurate diagnosis due to atypical clinical manifestations, negative sputum smear results, and a high incidence of peripheral tuberculosis. Additionally, research on tuberculosis biomarkers during pregnancy is relatively scarce, further increasing the difficulty of diagnosing pregnant tuberculosis patients with concurrent HIV infection. According to the WHO Tuberculosis Guidelines 2021, C-reactive protein is recommended as a screening biomarker for ambulatory HIV-infected individuals (WHO, 2021b). Besides the commonly used tuberculosis diagnostic methods (such as GeneXpert MTB/RIF, LAM, interferon-γ release assay (IGRA), tuberculin skin test (TST), smear, and culture), current research hotspots also include various immunobiomarkers (such as host serum proteins) and transcriptomics (Nogueira et al., 2022). Recent studies have pointed out that exosomes perform excellently in differentiating tuberculosis patients from non-tuberculosis patients, especially showing higher area under the curve values in peripheral tuberculosis, suggesting that these exosomal miRNAs may become potential diagnostic biomarkers for tuberculosis in HIV-infected individuals (Jin et al., 2024). However, most current research samples on HIV-tuberculosis infection are mainly focused on adults, and research on diagnostic biomarkers for children and pregnant patients remains insufficient.

The WHO recommends that HIV-infected mothers should receive combined antiretroviral therapy when their CD4 count is lower than 200 cells/mm³ and undergo routine tuberculosis screening before treatment (WHO, 2008). For patients diagnosed with active pulmonary tuberculosis concurrent with HIV, it is recommended that they receive combined antiretroviral (antiretroviral therapy, ART) and antituberculosis treatment regardless of CD4 cell count (Denebayeva et al., 2020). In addition, tuberculosis preventive treatment for HIV-infected individuals, including pregnant and lactating women, is also highly emphasized (Boyd et al., 2021). A case-control study from Thailand indicated that tuberculosis is an important risk factor affecting first-line ART. The reason is that *Mtb* can induce apoptosis of CD4+ cells, leading to CD4+ cell depletion, impairing the cellular immune response, and causing immune function exhaustion. In addition, the digestive burden of antiretroviral and antituberculosis drugs, adverse drug interactions, and side effects may result in poor patient tolerance and reduced compliance, thereby affecting the treatment outcome (Nasomsong et al., 2021). Hirsch-Moverman et al. (2024) surveyed tuberculosis screening and preventive treatment among pregnant and lactating women with AIDS in 44 countries. The results showed that 28 countries recommended tuberculosis screening for pregnant women with AIDS, and 35 countries (80%) recommended tuberculosis preventive treatment. However, compared with the AIDS pregnancy group, fewer countries recommended tuberculosis screening and preventive treatment for the AIDS breastfeeding group, only 32% (14 countries) and 45% (20 countries) respectively. Most countries recommend only isoniazid preventive treatment (IPT) for HIV-infected pregnant and lactating women, while some countries suggest the combination of isoniazid and rifampicin (3RH) or rifapentine (3HP).

Some studies have evaluated the safety and timing of IPT for HIV-infected pregnant women in a high-tuberculosis-burden environment. It was found that there was no significant difference in hepatotoxicity between women who received isoniazid treatment during pregnancy and those who received it after pregnancy. However, women who received IPT during pregnancy had an increased risk of adverse pregnancy outcomes (such as fetal death, preterm birth, low birth weight, and congenital anomalies). However, a sub-study showed that there was no correlation between IPT and adverse pregnancy outcomes (Gupta et al., 2019). Hamada et al. (2020) included 9 randomized controlled trials (RCTs) and non-randomized studies (NRS) of IPT for HIV-infected pregnant women and conducted a meta-analysis. The results showed that in one RCT, the risk of hepatotoxicity in pregnant women who received IPT increased (hazard ratio 1.64, 95% CI 0.78-3.44). Four of the studies compared the pregnancy outcomes of HIV-infected pregnant women with and without IPT exposure. It was found that in one RCT, adverse pregnancy outcomes were related to IPT exposure (OR 1.51, 95% CI 1.09-2.10), but three NRS showed a protective effect. Therefore, the association between IPT and adverse pregnancy outcomes is inconsistent.

In conclusion, tuberculosis increases women’s susceptibility to HIV and promotes the replication and transmission of HIV. Therefore, it is recommended to conduct routine tuberculosis screening and preventive antituberculosis treatment for HIV-infected individuals before starting antiretroviral treatment. For HIV-tuberculosis co-infected individuals, regardless of whether they are pregnant or not, they should receive combined antiretroviral and antituberculosis treatment, but the timing of treatment needs to be carefully evaluated by clinicians according to specific circumstances. For patients infected only with HIV, it is recommended to implement active tuberculosis preventive treatment (TPT). For HIV-infected women during pregnancy and lactation, IPT is recommended, but the specific prevention timing should be chosen by clinicians based on full consideration of the hepatotoxicity and adverse pregnancy outcomes that IPT may cause.

### 4.5 Tuberculosis and diabetes during pregnancy

Diabetes Mellitus (DM) is a chronic metabolic disorder characterized by elevated blood glucose levels (IDF, 2021). The estimated proportion of diabetic patients among global tuberculosis patients is 13.73% (Li et al., 2021). Pregnant women with both tuberculosis and diabetes face higher danger, such as the risks of preeclampsia, hypoglycemia, fetal preterm birth, and macrosomia (Rahmawati et al., 2022). Diabetes during pregnancy can be divided into two situations. One is diabetes that was diagnosed prior to pregnancy, referred to as “pregnancy complicated by diabetes”. The other occurs when a woman has normal blood glucose levels or potential impaired glucose tolerance before pregnancy, but develops or is diagnosed with diabetes only during gestation; this is known as gestational diabetes mellitus (GDM). Tuberculosis can trigger stress-related transient hyperglycemia. In most patients, fasting blood glucose will decrease significantly after antituberculosis treatment. However, about 38% of patients still have hyperglycemia six months after treatment, which emphasizes the importance of diabetes screening for tuberculosis patients (Aftab et al., 2017). But it should be noted that this conclusion was derived from studies on non-pregnant tuberculosis patients.

GDM exhibits insulin resistance similar to that of type 2 diabetes mellitus (T2DM). The maternal insulin resistance leads to hyperglycemia, and uncontrolled hyperglycemia promotes the transport of glucose in the placenta, which in turn stimulates fetal insulin secretion. Fetal insulin, as a growth-promoting hormone, can cause excessive growth of adipose tissue (such as around the chest, shoulders, and abdomen), increasing the risks of shoulder dystocia, perinatal death, and birth injury. The insulin resistance of most women will return to normal after childbirth, but about 20% of women will still have abnormal blood glucose levels after delivery (Alexander et al., 2019).

The global prevalence of diabetes has led to the number of tuberculosis patients with comorbid diabetes exceeding that of tuberculosis patients with comorbid HIV (Bodke et al., 2023). DM is an important risk factor for tuberculosis. The risk of developing tuberculosis in people with diabetes increases threefold (Jeon and Murray, 2008). In addition, the smear positivity rate of tuberculosis patients with comorbid diabetes is higher than that of non-diabetic patients. Compared with those who have only tuberculosis, the mortality rate of patients with tuberculosis accompanied by diabetes is twice that of the former (Kakisingi et al., 2024). Han Shi et al. (Siferih et al., 2024) found that patients with T2DMcomplicated by pulmonary tuberculosis (PTB) have poor blood glucose control and a higher infection frequency. Lymphopenia, smoking, a history of tuberculosis exposure, and poor blood glucose control are independent risk factors for T2DM-PTB, while overweight and obesity are considered protective factors. T2DM is a significant risk factor for the development of tuberculosis. In addition, some studies have pointed out that age is a risk-related factor for TB-DM comorbidities. The older the age, the higher the susceptibility to such comorbidities (Ekeke et al., 2017). Because of limited research on pregnant individuals with pulmonary tuberculosis and diabetes, findings from studies on non-pregnant individuals with diabetes and tuberculosis may have some reference value for pregnant populations.

A cohort study in India found that pregnant women with GDM complicated by HIV and latent tuberculosis had lower levels of interferon-γ (IFN-γ), interleukin-2 (IL-2), and interleukin-10 (IL-10) compared with women without GDM. In addition, another study also showed that interferon gamma release assay (IGRA) positive non-pregnant adults with T2DM also exhibited decreased levels of these cytokines (Faurholt-Jepsen et al., 2014; Chebrolu et al., 2022). Since these cytokines play an important role in the host’s immune response to *Mtb.* it is speculated that the immune response of diabetic patients to *Mtb* antigens is impaired. Among patients diagnosed with latent tuberculosis Infection (LTBI) using IGRA, the lower IFN-γ response in GDM patients may lead to the missed diagnosis of LTBI, causing them to miss the opportunity to receive tuberculosis preventive treatment. Moreover, due to the impaired immune response, the condition of these patients may progress to active pulmonary tuberculosis and even become more severe.

### 4.6 Tuberculosis, postpartum hemorrhage, anemia and sepsis in pregnant women

Postpartum hemorrhage and puerperal infection are important direct causes of maternal mortality, while anemia and tuberculosis are indirect causes (Gumanga et al., 2011; Desai et al., 2013; Singla et al., 2017). The WHO definition of postpartum hemorrhage is — blood loss of ≥500 ml within 24 hours after vaginal delivery, ≥1000 ml after cesarean section, or any blood loss accompanied by symptoms of hypovolemia (WHO, 2018). Anemia during pregnancy is defined as a peripheral blood hemoglobin (Hb) concentration of less than 110 g/L or a hematocrit (Hct) below 0.33 (Pranevicius et al., 2003). One study have pointed out that the causes of anemia in pregnant women with tuberculosis include specific bacillus intoxication, the inhibitory effects of antituberculosis drugs on the appetite and liver function of pregnant women, and insufficient absorption of nutrients (especially iron) (Pranevicius et al., 2003). In addition, anemia is also a clinical feature of pregnant women undergoing *in vitro* fertilization and embryo transfer (IVF-ET) complicated by miliary tuberculosis (Wang et al., 2022). Regarding sepsis, some studies have shown that Streptococcus pyogenes is the main pathogen (54.2%), and Mycobacterium tuberculosis accounts for 8.3% (Tanaka et al., 2019). A 2018 US retrospective cohort study found that mothers with tuberculosis had an 80% higher incidence of pregnancy complications compared to those without TB. These patients also showed increased rates of postpartum hemorrhage, sepsis, and anemia (Dennis et al., 2018). Another study pointed out that hospitalized mothers giving birth with tuberculosis were more likely to have postpartum anemia and require blood transfusions (El-Messidi et al., 2016). The study by Nguyen et al. (2022) showed that mothers with active tuberculosis had a higher risk of anemia, postpartum hemorrhage, and pregnancy-related death than those not infected.

In conclusion, pregnant women with tuberculosis infection have a significantly increased risk of postpartum hemorrhage, anemia, and sepsis, and these factors are all important direct or indirect causes of maternal death.

## 5 Discussion

The review indicates that mothers with tuberculosis have an increased risk of death and are more prone to complications such as hypertensive disorders in pregnancy, rupture of bullae, postpartum hemorrhage, sepsis, anemia, placental chorioamnionitis, and GDM. Pregnancy combined with tuberculosis infection also increases the risk of vertical transmission of HIV, raises the probability of newborns being infected with *Mtb*, and increases the possibility of the fetus having congenital tuberculosis. In addition, fetuses of mothers with pregnancy - combined tuberculosis face an increased risk of congenital malformations, intrauterine growth restriction, low birth weight, preterm birth, miscarriage, and stillbirth (**Table 1**). A 2024 retrospective cohort study in Malaysia shows that preterm birth, low birth weight, and small for gestational age infants are closely related to pregnant mothers with pulmonary tuberculosis, but have little relation to extrapulmonary tuberculosis (Zurina et al., 2024). Interestingly, One study have also pointed out that extrapulmonary tuberculosis limited to lymph nodes has no significant impact on pregnancy outcomes, while extrapulmonary tuberculosis in other sites has an adverse effect on pregnancy results (Jana et al., 1999).

**Table 1:** The summary of adverse pregnancy outcomes and complications of tuberculosis in pregnant women.

Study subject category	Adverse pregnancy outcomes and complications	Research type	Study population
Mother	Concurrent HIV, Chorioamnionitis, Preterm labor, Postpartum anemia, Blood transfusion, Pneumonia, Acute respiratory distress syndrome, Mechanical ventilation, Rise in maternal mortality	Retrospective cohort study (El-Messidi et al., 2016)	Pregnant women with tuberculosis
Infant	Congenital anomalies		
Mother	Rise in maternal morbidity, Anemia, Caesarean delivery	A meta-analysis (Sobhy et al., 2017)	Active tuberculosis in pregnancy
Infant	Preterm birth, Low birth weight, Birth asphyxia, Perinatal death		
Infant	Congenital TB	A meta-analysis (Snow et al., 2020)	Reviews on TB in pregnancy and newborns.
Mother	Anemia, Postpartum hemorrhage, Rise in maternal mortality	A meta-analysis (Nguyen et al., 2022)	Women with active tuberculosis
Infant	Low birth weight, Premature birth, Spontaneous or induced abortions		
Infant	Spina bifida and congenital hip dislocation	Retrospective cohort study (Pranevicius et al., 2003)	Women with tuberculosis
Infant	Congenital anomalies	A meta-analysis (Wu et al., 2023)	Anti-TB drugs usage during pregnancy.
Mother	Rupture of pulmonary bullae during pregnancy	Case report (Aziz and Suryawan, 2023)	Pregnancy with prior TB and giant lung bullae
Mother	Severe pre-eclampsia, Eclampsia, Placenta previa, Post-partum hemorrhage, Sepsis, Anemia, Hospital death	Retrospective cohort study (Dennis et al., 2018)	Tuberculosis during pregnancy in the United States
Mother	Acute chorioamnionitis	Case Report (Taweevisit et al., 2015)	Pregnant women with intrauterine tuberculosis
Mother	Preeclampsia, Hypoglycemia	Case report (Rahmawati et al., 2022)	Diabetes mellitus in pregnant women with tuberculosis
Infant	Preterm birth, Giant baby		
Infant	Congenital tuberculosis, Premature birth, Low birth weight, Small for gestational age, Low APGAR score	Retrospective cohort study (Zurina et al., 2024)	Pregnant mothers with pulmonary tuberculosis

Maternal outcomes are affected by multiple factors, including the severity of tuberculosis, the site of transmission, drug resistance, the timing of diagnosis and treatment, and comorbidities such as diabetes and HIV. Delayed diagnosis of PWT is the main cause of adverse pregnancy outcomes. Routine tuberculosis screening during pregnancy helps reduce diagnostic delay, thereby improving perinatal and maternal outcomes. Tuberculin skin tests or interferon - γ release assays can be used as safe screening methods for LTBI. Therefore, in order to prevent severe tuberculosis and related deaths and reduce the occurrence of adverse pregnancy outcomes, it is crucial to implement standardized whole - process management of PWT (Sobhy et al., 2017; Phoswa et al., 2020).

The strengths of this paper include a comprehensive summary of adverse pregnancy outcomes and complications of pulmonary tuberculosis during pregnancy, as well as the induction of management recommendations for complications. However, limitations exist: the summary predominantly focuses on patients with active pulmonary tuberculosis, with limited coverage of latent tuberculosis; specific treatment protocols for tuberculosis in pregnancy are not elaborated in detail. Based on a comprehensive literature review, the authors acknowledge persistent limitations in current research on tuberculosis in pregnancy: Failure to attribute adverse outcomes to tuberculosis, other diseases, or pregnancy itself; and Inability to determine causal drivers—whether outcomes originate from pre-pregnancy infection, pregnancy-associated infection, TB treatment, or site-specific infections. the authors emphasize that future research should shift toward precise analysis of the impact of tuberculosis infection sites, gestational age, and treatment status on outcomes, rather than merely focusing on whether tuberculosis is complicated with pregnancy. Additionally, the relationship between latent tuberculosis infection and pregnancy outcomes also requires enhanced research.
