# Supplementary material for: Adverse pregnancy outcomes and complications of tuberculosis in pregnant women
Source: Front Cell Infect Microbiol. 2025 Oct 2;15:1550430. doi: 10.3389/fcimb.2025.1550430 (PMC12528023; doi:10.3389/fcimb.2025.1550430)
Supplement: Supplementary file 1 [file Table1.docx]

Tuberculosis

Entry Terms:

- Tuberculoses
- Mycobacterium tuberculosis Infection
- Infection, Mycobacterium tuberculosis
- Infections, Mycobacterium tuberculosis
- Mycobacterium tuberculosis Infections
- Kochs Disease
- Koch's Disease
- Koch Disease

Pregnancy

Entry Terms:

• Pregnancies

• Gestation

**search query:**

（Pubmed 4,721 results，473page）(Web of Science 992 results)

("Pregnancy"[MeSH Terms] OR ("Pregnancy"[MeSH Terms] OR "Pregnancy"[All Fields] OR "pregnancies"[All Fields] OR "pregnancy s"[All Fields] OR ("gestate"[All Fields] OR "gestated"[All Fields] OR "gestates"[All Fields] OR "gestating"[All Fields] OR "gestational"[All Fields] OR "gestations"[All Fields] OR "Pregnancy"[MeSH Terms] OR "Pregnancy"[All Fields] OR "gestation"[All Fields]))) AND ("Tuberculosis"[MeSH Terms] OR ("tuberculosi"[All Fields] OR "Tuberculosis"[MeSH Terms] OR "Tuberculosis"[All Fields] OR "tuberculoses"[All Fields] OR "tuberculosis s"[All Fields] OR ("Tuberculosis"[MeSH Terms] OR "Tuberculosis"[All Fields] OR ("mycobacterium"[All Fields] AND "Tuberculosis"[All Fields] AND "infection"[All Fields]) OR "mycobacterium tuberculosis infection"[All Fields]) OR ("Tuberculosis"[MeSH Terms] OR "Tuberculosis"[All Fields] OR ("infection"[All Fields] AND "mycobacterium"[All Fields] AND "Tuberculosis"[All Fields]) OR "infection mycobacterium tuberculosis"[All Fields]) OR ("Tuberculosis"[MeSH Terms] OR "Tuberculosis"[All Fields] OR ("infections"[All Fields] AND "mycobacterium"[All Fields] AND "Tuberculosis"[All Fields]) OR "infections mycobacterium tuberculosis"[All Fields]) OR ("Tuberculosis"[MeSH Terms] OR "Tuberculosis"[All Fields] OR ("mycobacterium"[All Fields] AND "Tuberculosis"[All Fields] AND "infections"[All Fields]) OR "mycobacterium tuberculosis infections"[All Fields]) OR ("Tuberculosis"[MeSH Terms] OR "Tuberculosis"[All Fields] OR ("kochs"[All Fields] AND "disease"[All Fields]) OR "kochs disease"[All Fields]) OR ("Tuberculosis"[MeSH Terms] OR "Tuberculosis"[All Fields] OR ("koch s"[All Fields] AND "disease"[All Fields]) OR "koch s disease"[All Fields]) OR ("Tuberculosis"[MeSH Terms] OR "Tuberculosis"[All Fields] OR ("koch"[All Fields] AND "disease"[All Fields]) OR "koch disease"[All Fields])))

Translations

Pregnancies: "pregnancy"[MeSH Terms] OR "pregnancy"[All Fields] OR "pregnancies"[All Fields] OR "pregnancy's"[All Fields]

Gestation: "gestate"[All Fields] OR "gestated"[All Fields] OR "gestates"[All Fields] OR "gestating"[All Fields] OR "gestational"[All Fields] OR "gestations"[All Fields] OR "pregnancy"[MeSH Terms] OR "pregnancy"[All Fields] OR "gestation"[All Fields]

Tuberculoses: "tuberculosi"[All Fields] OR "tuberculosis"[MeSH Terms] OR "tuberculosis"[All Fields] OR "tuberculoses"[All Fields] OR "tuberculosis's"[All Fields]

Mycobacterium tuberculosis Infection: "tuberculosis"[MeSH Terms] OR "tuberculosis"[All Fields] OR ("mycobacterium"[All Fields] AND "tuberculosis"[All Fields] AND "infection"[All Fields]) OR "mycobacterium tuberculosis infection"[All Fields]

Infection, Mycobacterium tuberculosis: "tuberculosis"[MeSH Terms] OR "tuberculosis"[All Fields] OR ("infection"[All Fields] AND "mycobacterium"[All Fields] AND "tuberculosis"[All Fields]) OR "infection, mycobacterium tuberculosis"[All Fields]

Infections, Mycobacterium tuberculosis: "tuberculosis"[MeSH Terms] OR "tuberculosis"[All Fields] OR ("infections"[All Fields] AND "mycobacterium"[All Fields] AND "tuberculosis"[All Fields]) OR "infections, mycobacterium tuberculosis"[All Fields]

Mycobacterium tuberculosis Infections: "tuberculosis"[MeSH Terms] OR "tuberculosis"[All Fields] OR ("mycobacterium"[All Fields] AND "tuberculosis"[All Fields] AND "infections"[All Fields]) OR "mycobacterium tuberculosis infections"[All Fields]

Kochs Disease: "tuberculosis"[MeSH Terms] OR "tuberculosis"[All Fields] OR ("kochs"[All Fields] AND "disease"[All Fields]) OR "kochs disease"[All Fields]

Koch's Disease: "tuberculosis"[MeSH Terms] OR "tuberculosis"[All Fields] OR ("koch's"[All Fields] AND "disease"[All Fields]) OR "koch's disease"[All Fields]

Koch Disease: "tuberculosis"[MeSH Terms] OR "tuberculosis"[All Fields] OR ("koch"[All Fields] AND "disease"[All Fields]) OR "koch disease"[All Fields]
